# Coronavirus disease 2019 (COVID-19) in children: a systematic review of imaging findings

**DOI:** 10.1007/s00247-020-04726-w

**Published:** 2020-06-18

**Authors:** Susan C. Shelmerdine, Jovan Lovrenski, Pablo Caro-Domínguez, Seema Toso, Collaborators of the European Society of Paediatric Radiology Cardiothoracic Imaging Taskforce, Efi Alexopoulou, Judith Almanza, Alistair D. Calder, Pierluigi Ciet, Beatrice Damasio, Sarah M. Desoky, David Gomez-Pastrana, Hyun Woo Goo, Sureyya Burcu Gorkem, Franz Wolfgang Hirsch, Christian Kellenberger, Maryam Ghadimi Mahani, Maria Navallas, Catherine M. Owens, Maria Raissaki, Lucia Riaza, Rick R. van Rijn, Joost van Schuppen, Aurelio Secinaro, Paolo Toma, Carlos S. Ugas Charcape

**Affiliations:** 1 Department of Radiology, Great Ormond Street Hospital for Children, Great Ormond Street, London, WC1N 3JH UK; 2 UCL Great Ormond Street Institute of Child Health, Great Ormond Street Hospital for Children, London, UK; 3 Great Ormond Street Hospital NIHR Biomedical Research Centre, London, UK; 4 Faculty of Medicine, University of Novi Sad, Novi Sad, Serbia; 5 Institute for Children and Adolescents Health Care of Vojvodina, Novi Sad, Serbia; 6 Unidad de Radiología Pediátrica, Servicio de Radiodiagnóstico, Hospital Universitario Virgen del Rocío, Sevilla, Spain; 7 Department of Diagnostics, Pediatric Radiology, Geneva Children’s Hospitals, Geneva, Switzerland

**Keywords:** Adolescents, Children, Computed tomography, Coronavirus, COVID-19, Imaging, Radiology, Systematic review

## Abstract

**Background:**

COVID-19 is a novel coronavirus infection that can cause a severe respiratory illness and has been declared a pandemic by the World Health Organization (WHO). Because children appear to be less severely affected than adults, their imaging appearances have not been extensively reported.

**Objective:**

To systematically review available literature regarding imaging findings in paediatric cases of COVID-19.

**Materials and methods:**

We searched four databases (Medline, Embase, Cochrane, Google Scholar) for articles describing imaging findings in children with COVID-19. We included all modalities, age <18 years, and foreign language articles, using descriptive statistics to identify patterns and locations of imaging findings, and their association with outcomes.

**Results:**

Twenty-two articles were included, reporting chest imaging findings in 431 children, of whom 421 (97.7%) underwent CT. Criteria for imaging were lacking. At diagnosis, 143/421 (34.0%) had a normal CT. Abnormalities were more common in the lower lobes and were predominantly unilateral. The most common imaging pattern was ground-glass opacification (159/255, 62.4%). None of the studies described lymphadenopathy, while pleural effusions were rare (three cases). Improvement at follow-up CT imaging (3–15 days later) was seen in 29/100 (29%), remained normal in 25/100 (25%) and progressed in 9/100 (9%).

**Conclusion:**

CT chest findings in children with COVID-19 are frequently normal or mild. Lower lobes are predominantly affected by patchy ground-glass opacification. Appearances at follow-up remain normal or improve in the majority of children. Chest CT imaging adds little to the further management of the patient and should be reserved for severe cases or for identifying alternative diagnoses.

**Electronic supplementary material:**

The online version of this article (10.1007/s00247-020-04726-w) contains supplementary material, which is available to authorized users.

## Introduction

A novel strain of coronavirus (referred to as 2019-nCoV or SARS-CoV-2), which causes the sometimes severe respiratory infection COVID-19, was first identified in Wuhan city, China, toward the end of 2019 [1]. By 12 March 2020, COVID-19 was declared a global pandemic by the World Health Organization (WHO), and at the time of writing it had spread to 187 countries, with almost 3.5 million confirmed cases, and had claimed more than 244,000 lives [2].

Epidemiological studies originating from China have shown that children are less likely to be clinically affected than older adults, with one study finding only 0.9% of those affected being younger than 15 years old [3]. A different Chinese study, which included 731 confirmed paediatric cases, found that the majority (84.1%) sustained either mild or moderate symptoms, with <3% described as being severely or critically affected [4]. Only one study reported a death in a child, a 10-month-old with multiorgan failure and intussusception [5]. In the United States, data published on 2 April 2020 [6] showed that only 1.7% (*n*=2,572) of people affected by COVID-19 were younger than 18 years, with 0.58–2% of children requiring intensive care admission. Given the low number of paediatric cases, keeping abreast of the latest information and assimilating the combined knowledge of radiographic findings in infected children is challenging. Whilst several systematic reviews of imaging findings in COVID-19 cases have been performed for adults [7, 8], none has specifically focused on children.

The overall objective of this study was therefore to assimilate the available information on imaging features of COVID-19 disease in children. Particular points of interest include identifying typical imaging findings during diagnosis and follow-up stages of the infection, and whether any features might be used as prognostic markers to determine patient outcome. Where knowledge gaps exist, we intend to highlight these and suggest potential future avenues for research.

## Materials and methods

Ethics approval was not required for this retrospective review of published data. We followed Preferred Reporting Items for Systematic reviews and Meta-Analyses (PRISMA) guidelines for transparent reporting of systematic reviews. This study was registered in PROSPERO, an international prospective register of systematic reviews (Registration ID: CRD42020175945 [9]).

### Literature review

A systematic literature search was performed of Medline (Ovid), Embase and the Cochrane Library databases for the latest articles published between 1 January 2015 and 17 March 2020 (5-year range), using database-specific Boolean search strategies with key terms and word variations relating to all three categories:

1. “coronavirus”, “COVID-19”, “SARS-CoV-2” or “2019-nCoV”;
2. “paediatrics”, “children”, “neonate”, “infant” or “adolescent”;
3. “radiology”, “imaging”, “ultrasound”, “CT”, “MRI” or “radiography.”

Full search terms are shown in the supplementary material (Tables S1, S2 and S3). To include as many recent articles as possible, we also performed a grey literature search (i.e. literature not formally published in sources such as books or journal articles [10], such as government white paper articles and guidelines) using the same keywords on Google Scholar and for any WHO Global Library publications. Additional articles were retrieved by manual screening of the reference lists of included studies and relevant review articles/editorial pieces. The initial search was conducted on 17 March 2020. A repeat search was conducted on 23 March 2020, and again on 30 April 2020 for any further eligible manuscripts.

### Eligibility criteria

Inclusion criteria encompassed all studies investigating and describing imaging findings of confirmed COVID-19 infection in children, using reverse transcriptase polymerase chain reaction (RT-PCR) testing. Studies were limited to human subjects, including foetuses (any gestation) and children (ages 0–18 years). No restrictions were placed on type of imaging modality, number of cases described or type of clinical setting. To widen our search and include as many cases as possible, we included case reports. No language restrictions were used given that many early articles on imaging findings have been published in Chinese. Where this was the case, we sought online translation services and advice from native-speaking colleagues to interpret reported findings.

Exclusion criteria included studies reported as editorials, opinion articles or multimedia files (online videos, podcasts). Suspected but unconfirmed cases of COVID-19 were not included. Studies relating to other coronavirus-related illnesses, such as Middle East respiratory syndrome (MERS) or severe acute respiratory syndrome (SARS) were excluded. We also excluded any articles reporting on a mixed adult and paediatric cohort where imaging results for the paediatric cohort could not be extracted.

### Data extraction and quantitative data synthesis

All articles were independently searched by two reviewers (S.C.S. and S.T. with 7 years and 10 years of paediatric radiology experience, respectively). They examined abstracts of suitable studies and obtained full papers according to the eligibility criteria. Disagreements were resolved by consensus.

The same two reviewers (S.C.S., S.T.) independently extracted data from the full articles into a database (Excel; Microsoft, Redmond, WA), which included the following factors: study design, study setting/country, population demographics (e.g., gender, age, underlying comorbidities), sample size, patient outcomes (number of mortalities), imaging modality and imaging findings (pattern and location of involvement of disease) and results of any follow-up imaging.

Missing data were recorded as “not recorded” or “not stated”. Authors of published studies were not contacted because of the tight time constraints involved in the completion of the systematic review during the unprecedented time of need for this information.

### Methodological quality

The quality for each included study was assessed using the National Institutes of Health Quality Assessment Tool for Case Series Studies [11] by two reviewers (S.T. and J.L, with 17 years of paediatric radiology experience). Disagreements were resolved by consensus review. Any that could not be resolved by consensus was arbitrated by a third reviewer (S.C.S.).

### Statistical analysis

We planned a meta-analysis to assess association of imaging findings with patient outcomes and demographic data; however, we omitted this because of a lack of sufficient data, with many cases being incompletely reported. Therefore, we used descriptive statistics to determine frequency and percentages of imaging appearances across different articles.

## Results

### Included studies

During the initial literature search, after removing duplicates, we identified 146 unique records. After screening titles and abstracts, we excluded 100 studies and checked 46 full-text articles. Reasons for exclusions included insufficient description regarding imaging results (*n*=8), opinion pieces (*n*=7), adult population only (*n*=5), no confirmed (only suspected) COVID-19 cases (*n*=3), retracted article (*n*=2) and no full text available (*n*=1). After the second and third searches of the databases, we found eight more records that met our inclusion criteria and reviewed them.

Although it was not explicitly stated in the articles, we found that a small case series of 8 children [12] and a larger one of 115 children [13] appeared to describe a subset of a larger case series already published of 171 children with COVID-19 [5] (included in our review). All three articles originated from Wuhan city, China. We therefore excluded the two smaller studies from further qualitative or quantitative analysis. We have provided the reference and summary results from the smaller articles in our supplementary appendix (Tables S4 and S5; [5, 12–34]) to alert readers to this overlap and show why we reached this conclusion.

### Methodological quality

Most articles were given an overall scoring of “fair” (17/26, 65.4%), with 5/26 (19.2%) described as “good”. Four of 26 (15.4%) articles were scored as “poor”; these were excluded from further analysis, mainly for poor descriptions of radiographic findings in non-representative cohorts [35–38]. Methodological quality assessment of the studies is presented in Table 1 [5, 13–22, 24–34, 36–38].

**Table 1:** Quality ratings of included studies according to the National Institutes of Health (NIH) Quality Assessment Tool for Case Series Studies [11]

Author [reference]	Question^a^											Final consensus
	Reviewer	1	2	3	4	5	6	7	8	9	Overall rating	
Cai J et al. [14]	1	N	Y	N	CD	NA	Y	CD	NA	Y	Fair	Fair
	2	Y	Y	Y	NA	N	Y	CD	Y	Y	Fair	
Chan JF et al. [15]	1	Y	Y	NR	CD	NA	Y	CD	NA	Y	Fair	Fair
	2	Y	Y	Y	Y	N	Y	CD	Y	Y	Fair	
Chen C et al. [16]	1	Y	Y	NR	NA	N	Y	N	Y	Y	Fair	Fair
	2	Y	Y	Y	CD	N	Y	CD	Y	Y	Fair	
Chen F et al. [35]	1	N	NA	NA	NA	N	Y	CD	NA	N	Fair	Poor
	2	N	Y	NA	NA	N	NA	CD	NA	Y	Poor	
Cui Y et al. [17]	1	Y	NA	NA	CD	Y	Y	Y	NA	Y	Good	Good
	2	Y	Y	NA	CD	Y	NA	Y	NA	Y	Good	
Feng K et al. [18]	1	Y	Y	NR	CD	Y	Y	Y	N	Y	Fair	Fair
	2	Y	Y	CD	CD	Y	Y	CD	N	Y	Fair	
Hu Z et al. [19]	1	Y	Y	NR	CD	N	Y	Y	Y	Y	Fair	Fair
	2	Y	Y	CD	Y	N	Y	Y	Y	Y	Good	
Ji LN et al. [20]	1	Y	Y	NR	CD	N	Y	CD	NA	Y	Fair	Fair
	2	Y	Y	NA	CD	N	Y	CD	NA	Y	Good	
Li W et al. [21]	1	Y	Y	NR	CD	N	Y	Y	NA	Y	Fair	Fair
	2	Y	Y	CD	CD	N	Y	Y	NA	Y	Good	
Liu H et al. [22]	1	Y	Y	NR	Y	Y	Y	N	Y	Y	Good	Good
	2	Y	Y	CD	CD	Y	Y	CD	Y	Y	Good	
Liu M et al. [31]	1	Y	N	NR	CD	N	Y	CD	NA	Y	Fair	Fair
	2	Y	Y	NR	Y	Y	N	Y	NA	N	Poor	
Lu X et al. [5]	1	Y	Y	NR	CD	N	Y	N	N	Y	Fair	Fair
	2	Y	Y	CD	CD	N	Y	CD	N	Y	Good	
Ma H et al. [23]	1	Y	Y	NR	CD	Y	Y	Y	Y	Y	Good	Good
	2	Y	Y	CD	CD	Y	Y	Y	Y	Y	Good	
Pan X et al. [24]	1	N	Y	NA	CD	N	NA	N	NA	Y	Fair	Fair
	2	N	Y	Y	Y	N	Y	Y	NA	Y	Fair	
Park JY et al. [25]	1	Y	NA	NA	NA	N	Y	N	NA	Y	Fair	Fair
	2	Y	NA	NA	NA	N	Y	Y	NA	Y	Fair	
Qiu H et al. [30]	1	Y	Y	Y	NA	N	Y	CD	Y	Y	Fair	Fair
	2	N	Y	NR	Y	Y	Y	NR	NA	N	Poor	
Rahimzadeh G et al. [29]	1	N	Y	NR	CD	Y	Y	Y	N	Y	Fair	Fair
	2	Y	Y	CD	CD	N	Y	CD	N	Y	Fair	
Shen Q et al. [32]	1	Y	Y	NA	Y	Y	Y	Y	NA	Y	Fair	Fair
	2	Y	Y	NR	Y	Y	Y	Y	NA	Y	Fair	
Tang A et al. [26]	1	Y	Y	NR	CD	N	Y	CD	Y	Y	Fair	Fair
	2	Y	Y	CD	CD	N	Y	CD	Y	Y	Fair	
Wang D et al. [36]	1	Y	Y	NR	CD	N	Y	CD	N	N	Fair	Poor
	2	Y	N	CD	CD	N	N	CD	N	N	Poor	
Wang S et al. [27]	1	Y	NA	NA	NA	N	Y	Y	NA	Y	Good	Good
	2	Y	NA	NA	NA	N	NA	Y	NA	Y	Good	
Xia W et al. [28]	1	Y	Y	NR	CD	Y	Y	Y	N	Y	Good	Good
	2	Y	Y	Y	CD	Y	Y	CD	N	Y	Good	
Zeng LK et al. [37]	1	N	NA	NA	NA	N	Y	CD	NA	N	Fair	Poor
	2	N	NA	NA	NA	N	Y	CD	NA	N	Poor	
Zhang Y et al. [38]	1	N	NA	NA	NA	N	Y	Y	NA	N	Poor	Poor
	2	N	Y	NA	NA	N	Y	CD	NA	N	Poor	
Zheng F et al. [33]	1	N	Y	Y	Y	Y	Y	N	NA	Y	Fair	Fair
	2	N	Y	Y	Y	Y	Y	N	Y	Y	Fair	
Zhou Y et al. [34]	1	Y	Y	NR	NA	Y	Y	Y	NA	Y	Fair	Fair
	2	Y	Y	NR	Y	Y	Y	Y	NA	N	Fair	

*CD* cannot determine, *N* no, *NA* not applicable, *NR* not reported, *Y* yes

^a^The NIH Quality Assessment Tool for Case Series Studies questions include the following nine questions: (1) Was the study question or objective clearly stated?; (2) Was the study population clearly and fully described, including a case definition?; (3) Were the cases consecutive?; (4) Were the subjects comparable?; (5) Was the intervention (i.e. imaging modality) clearly described?; (6) Were the outcome measures clearly defined, valid, reliable, and implemented consistently across all study participants?; (7) Was the length of follow-up adequate?; (8) Were the statistical methods well-described?; and (9) Were the results well-described?

We therefore analysed 22 studies overall in this systematic review (19 case series, 3 case reports), including 431 children with imaging (241 male, or 55.9%) [5, 12, 14, 15, 17–34, 39]. Two (2/22, 9.1%) articles were in Chinese [18, 34], the remainder in English. All articles were published in 2020, over a 4-month period from January to April.

### Patient demographics

The demographics of the children and their presenting complaints are summarised in Table 2 [5, 14–34] and expanded upon in the supplementary material (Table S4). All children were confirmed COVID-19 cases on RT-PCR testing. The studies mainly originated from China (20/22, 91.0%), with 1/22 (4.5%) from South Korea [25] and 1/22 (4.5%) from Iran [29]. Of the Chinese studies, 4/22 (18.2%) described cases from Wuhan city [5, 23, 27, 28]. The youngest child in our cohort was 36 h old; the oldest was 17 years old.

**Table 2:** Initial imaging characteristics of children with COVID-19

Author	Sample size (children)	Imaging modality	Initial imaging timing^a^	Initial imaging findings^b^
Cai J et al. [14]	10	CXR	Admission	6 (60%) normal 4 (40%) unilateral patchy infiltrates (1 retrocardiac opacity, left lung; 3 right lung opacification)
Chan JF et al. [15]	1	Chest CT	NS	CT showed GGO (location and laterality not mentioned)
Chen C et al. [16]	31	Chest CT	Admission	20 (64.5%) normal 8 (25.8%) unilateral pneumonia 3 (9.7%) bilateral pneumonia Example imaging in 4 cases all demonstrated patchy peripheral GGO in middle or lower lobes
Cui Y et al. [17]	1	Chest CT	Admission (Day 6 post symptoms)	Mild perihilar GGO in RUL and RLL
Feng K et al. [18]	15	Chest CT	Admission	6 (40%) normal 9 (60) inflammatory infiltrations — patchy nodular, GGO, visible halo sign (4 in single lobar segment; 4 in at least two lobar segments; 1 in more than two lobar segments)
Hu Z et al. [19]	6	Chest CT	Admission	4 (66.7%) normal 1 (16.7%) right basal subpleural ground-glass opacification 1 (16.7%) ground-glass opacification/patchy shadowing
Ji LN et al. [20]	2	Chest CT	NS	2 (100%) normal
Li W et al. [21]	5	Chest CT	4 days post admission (range 2–9 days)	2 (40%) normal 3 (60%) patchy GGO (2 LLL, 1 RUL)
Liu H et al. [22]	4	Chest CT	NS, presumed on admission	1 (25%) normal 1 (25%) single area of consolidative change, RLL 1 (25%) single area of GGO, RML 1 (25%) multifocal consolidation + pleural effusion
Liu M et al. [31]	5	Chest CT	Admission	1 (20%) normal 2 (40%) unilateral GGO 1 (204%) unilateral GGO and consolidation 1 (20%) bilateral GGO
Lu X et al. [5]	171	Chest CT	NS	60 (35%) none 56 (32.7%) ground-glass opacification 32 (18.7%) local patchy shadowing 21 (12.3%) bilateral patchy shadowing 2 (1.2%) interstitial abnormalities
Ma H et al. [23]	50	Chest CT	NS, presumed on admission	29 (67%) ground-glass opacities 16 (37%) localized patchy shadowing 9 (21%) bilateral patchy shadows 3 (7%) interstitial lesions 1 (2%) pleural effusion Lesion location 28 (65%) lower lobe 9 (18%) middle lobe 22 (44%) upper lobe
Pan X et al. [24]	1	Chest CT	1 day post admission	Normal
Park JY et al. [25]	1	CXR Chest CT	Admission CXR and CT	CXR: normal CT: patchy, nodular consolidations with peripheral GGO in subpleural areas of RLL
Qiu H et al. [30]	36	Chest CT	NS	17 (47%) normal 19 (53%) GGO
Rahimzadeh G et al. [29]	3 RT-PCR positive cases	CXR Chest CT	CXR at admission CT not specified, presumed upon admission also	CXR 2 (66.7%) ‘airspace shadowing’ (location not specified) 1 (33.3%) no findings recorded, uncertain if this was performed CT 2 (66.7%) patchy consolidation with halo sign, and GGO in both lungs 1 (33.3%) normal
Shen Q et al. [32]	9	CXR Chest CT	NS — both modalities reported together in report	7 (77.8%) normal 2 (22.2%) unilateral GGO
Tang A et al. [26]	26	CXR Chest CT	NS	11 (42%) lateral pulmonary infiltrates 7 (27%) bilateral pulmonary infiltrates
Wang S et al. [27]	1	CXR Chest CT	CXR at Day 2 post admission CT at Day 4 post admission	Initial CXR showed thickened lung texture Initial CT showed high-density nodular shadow at posterior segment of LUL
Xia W et al. [28]	20	Chest CT	NS, presumed on admission	4 (20%) normal 6 (30%) unilateral pulmonary lesion 10 (50%) bilateral lung lesions Of these, 16 (80%) subpleural ground-glass opacities 10 (50%) central consolidation with surrounding ground-glass halo 12 (60%) ground-glass opacities 4 (20%) fine mesh shadow 3 (15%) micronodules
Zheng F et al. [33]	25	Chest CT (in 24/25, 96% cases)	NS, presumed on admission	8 (33%) normal 5 (21%) unilateral findings 11 (46%) bilateral findings Number of cases with different pattern of abnormalities not stated, although typical findings of bilateral patchy shadows or consolidations were mentioned
Zhou Y et al. [34]	9	Chest CT	CT within 3 days of admission	Pattern 1 (11.1%) normal (incidental bullae noted in LLL) 8 (88.9%) inflammatory changes (6 GGO with consolidation; 1 consolidation only; 1 GGO only) 1 (11.1%) pleural effusion 3 (33.3%) halo sign Distribution 4 (50%) bilateral 4 (50%) unilateral Location 6 (75%) upper lobe 6 (75%) lower lobe 5 (62.5%) middle lobe

^a^Initial timing of imaging might be days since onset of symptoms or days since admission to hospital

^b^Imaging patterns described are as written in the publications

*CT* computed tomography, *CXR* chest radiography, *GGO* ground-glass opacification, *LLL* left lower lobe, *LUL* left upper lobe, *NC* non-contrast, *NS* not stated, *RLL* right lower lobe, *RML* right middle lobe, *RT-PCR* reverse transcriptase polymerase chain reaction, *RUL* right upper lobe, *US* ultrasound

Ninety-eight (98/431, 22.7%) children were asymptomatic upon admission to hospital, being assessed for having recent travel to a COVID-19 endemic area or close contact with an infected individual. Of those with symptoms, fever (120/333, 36.0%) and coughing (139/333, 41.7%) were the more common presenting complaints.

### Imaging modality and parameters

The majority of the imaging modalities described in the studies were chest CT (21/22, 95.5%), of which only 1/21 (4.8%) utilised intravenous contrast agent for imaging [25]. In one study (1/22, 4.5%) only chest radiography findings were described [14]. In 5/22 (22.7%) studies, a combination of both chest radiography and CT imaging findings were described [25–27, 29, 32]. None of the studies described MRI, nuclear medicine or chest ultrasound findings (see supplementary material for full details, Table S5 [5, 12–34]).

In only 5/22 (22.7%) studies were the CT vendor and scanner model described [18, 22, 23, 28, 34]. Of these, four articles provided detailed imaging acquisition parameters [18, 22, 28, 34]. One study did not report on the CT scanner model but did report upon the imaging parameters [17]. Details on imaging protocols and parameters are provided in the supplementary material, Table S6 [5, 15–34]. In only 10/22 (45.5%) articles was a radiologist identified as a co-author. Detailed indications for performing CT imaging were lacking in all studies.

### Initial imaging findings

Where CT imaging was used, 143/421 (34.0%) cases did not have any radiographic findings despite being COVID-19 positive. In the one study where only chest radiography was used, 6/10 (60%) of the imaged cases were normal [14]. In a case report from South Korea, the admission chest radiography was normal, although the CT demonstrated patchy nodular consolidation with ground-glass opacification [25].

Throughout all studies, there was significant heterogeneity in terminologies used with reference to radiographic findings, many of which included well-known terms (e.g., ground-glass opacifications) as well as non-standard descriptive terminologies (e.g., “thickened lung texture” [27]). In addition, missing information regarding imaging findings made summarising these difficult. Despite this, available findings are summated Tables 3 and 4 [5, 14–34]. It should be acknowledged that the percentage of cases for the various descriptors is less important than their relative frequencies to one another, given the missing information.

**Table 3:** Summarised initial imaging characteristics of children with COVID-19

Author [reference]	Sample size	Imaging modality	Abnormalities		Lobe affected				Laterality		Segments		Subpleural
			None	Present	UL	ML/ lingula/ hilar	LL	Multi-lobar/ diffuse	Unilateral	Bilateral	One	Two	
Cai J et al. [14]	10	CXR	6	4					4				
Chan JF et al. [15]	1	Chest CT	0	1									
Chen C et al. [16]	31	Chest CT	20	11					8	3			
Cui Y et al. [17]	1	Chest CT	0	1	1		1		1				
Feng K et al. [18]	15	Chest CT	6	9				1			4	4	
Hu Z et al. [19]	6	Chest CT	4	2			1		1				1
Ji LN et al. [20]	2	Chest CT	2	0									
Li W et al. [21]	5	Chest CT	2	3	1		2						
Liu H et al. [22]	4	Chest CT	1	3		1	1	1					
Liu M et al. [31]	5	Chest CT	1	4					3	1			
Lu X et al. [5]	171	Chest CT	60	111					32	21			
Ma H et al. [23]	50	Chest CT	0	50	22	9	28			9			
Pan X et al. [24]	1	Chest CT	1	0									
Park JY et al. [25]	1	CXR Chest CT	0	1			1		1				1
Qiu H et al. [30]	36	Chest CT	17	19									
Rahimzadeh G. et al. [29]	3	CXR Chest CT	1	2				2		2			
Shen Q et al. [32]	9	CXR Chest CT	7	2					2				
Tang A et al. [26]	26	CXR Chest CT	8	18					11	7			11
Wang S et al. [27]	1	CXR Chest CT	0	1	1								
Xia W et al. [28]	20	Chest CT	4	16					6	10			
Zheng F et al. [33]	25 (24 had CT)	Chest CT	8	16					5	11			
Zhou Y et al. [34]	9	Chest CT	1	8	6	5	6		4	4			
Total	431	-	149/431 (34.6%)	282/431 (65.4%)	31/90 (34.4%)	15/90 (16.7%)	40/90 (44.4%)	4/90 (4.4%)	78/146 (53.4%)	68/146 (46.6%)	4	4	13

The findings correspond to readily available reported imaging findings within the relevant publications. Only COVID-19 confirmed cases by reverse transcriptase polymerase chain reaction (RT-PCR) are included. Because of the heterogeneous and occasionally incomplete reporting of these findings (e.g., some without pathology location, some using different terminologies), not all features are mutually exclusive, nor total to the combined number of patients; therefore, percentages are not provided for features that were only reported by a small number of publications (e.g., segmental involvement and subpleural distribution), and the denominator for lobe affected and laterality is derived for total number of studies where these findings were stated

*CT* computed tomography, *CXR* chest radiograph, *LL* lower lobe, *ML* middle lobe, *UL* upper lobe

**Table 4:** Patterns of imaging findings on initial CT study in children with COVID-19

Author [reference]	Abnormal CT (*n*)	Pattern described (*n*)	Pattern						
			GGO	Consolidation	“Halo sign”	Pulmonary “infiltrates/ shadows”	Interstitial lesions	Nodular appearances	Pleural effusion
Chan JF et al. [15]	1	1	1						
Chen C et al. [16]	11	4	4						
Cui Y et al. [17]	1	1	1						
Feng K et al. [18]	9	9	9		9	9			
Hu Z et al. [19]	2	2	2			1			
Li W et al. [21]	3	3	3						
Liu H et al. [22]	3	3	1	2					1
Liu M et al. [31]	4	4	4	1					
Lu X et al. [5]	111	111	56			53	2		
Ma H et al. [23]	50	50	29			25	3		1
Park JY et al. [25]	1	1	1	1					
Qiu H et al. [30]	19	19	19						
Rahimzadeh G et al. [29]	2	2	2	2	2				
Shen Q et al. [32]	2	2	2						
Tang A et al. [26]	18	18				18			
Wang S et al. [27]	1	1	1			1			
Xia W et al. [28]	16	16	16	1	10	4		3	
Zhou Y et al. [34]	8	8	8	7	3				1
Total	262	255	159 (62.3%)	14 (5.5%)	24 (9.4%)	111 (43.5%)	5 (1.9%)	4 (1.6%)	3 (1.2%)

Descriptors refer to those stated within the relevant publications. Because of the heterogeneous, non-standard terminologies, we included descriptions of “shadows/infiltrates” together and interpret these to mean nonspecific opacities. In many articles, there was incomplete reporting of findings; therefore, not all features are mutually exclusive, nor total to the combined number of children within the study. The column titled “pattern described” is therefore included to demonstrate how many of the reported abnormal CT cases for which the study provided the abnormalities. The relative frequencies of findings are provided (with the “total pattern described” as denominator, rather than total abnormal CT), which is the more important indicator than the absolute numbers summated

*CT* computed tomography, *GGO* ground-glass opacification

From the reports where location of pathology was stated, this was identified in the upper lobe in 31/90 (34.4%), middle lobe in 15/90 (16.7%) and the lower lobe of the lung in 40/90 (44.4%) children. Diffuse/multifocal disease was described in 4 patients (4.4%).

Pathology was unilateral in 78/146 (53.4%) and bilateral in 68/146 (46.6%) children. Subpleural disease (i.e. peripheral in location) was specifically described in 13 cases across three studies [19, 25, 26].

Where a specific pattern was described, the most characteristic pattern on CT was ground-glass opacity, reported in 159/255 (62.3%) children. Patchy consolidations were seen in 14/255 (5.5%) patients. A so-called halo sign (of ground-glass opacification) around areas of consolidation was reported in 24/255 (9.4%) children across four studies [18, 28, 29, 34]. Nonspecific terminologies of “lung infiltrates/shadows” were reported in 111/255 (43.5%) and interstitial lesions in 5/255 (1.9%) children. Not all articles had case-specific individual descriptions of chest radiography and CT findings, which were frequently described together.

Several findings were either not reported or only rarely reported. For example, none of the articles described significant mediastinal lymphadenopathy or cavitation on imaging, although only one study performed a contrast-enhanced CT. In only 3/255 (1.2%) children there were pleural effusions. Of these, one was a neonate also infected with respiratory syncytial virus (RSV) [22]. The underlying conditions of the other cases were not reported [23].

### Follow-up imaging findings

In 11/22 (50%) studies, repeat CT imaging results were described, representing a total of 100 children. The CT was performed 3–15 days after admission, with a quarter of imaging studies remaining normal (25/100, 25%) or showing some improvement from previously detected abnormalities (29/100, 29%). In a minority of cases the abnormalities had progressed (9/100, 9%) or new findings developed (4/100, 4%). Findings were stable in 18/100 (18%) cases, and a complete resolution of previous abnormalities was seen in 15/100 (15%) (Table 5) [16–19, 21–23, 27, 28, 31, 34]. For one study where only chest radiography follow-up was performed, findings remained normal [25].

**Table 5:** Follow-up CT imaging findings in children with COVID-19

Author [reference]	Cases with follow-up imaging	Timing post admission (days)	Remained normal	Complete resolution of abnormalities	Improving abnormalities	Abnormalities unchanged	New abnormalities (previously normal)	Progressive changes
Chen C et al. [16]	28	NS	19			7	1	1
Cui Y et al. [17]	1	11			1			
Feng K et al. [18]	15	3–5	3	2		7	3	
Hu Z et al. [19]	1	13		1				
Li W et al. [21]	3	5–7		3				
Liu H et al. [22]	3	7	1		2			
Liu M et al. [31]	5	4–14	1	4				
Ma H et al. [23]	29	NS		2	17	2		8
Wang S et al. [27]	1	15			1			
Xia W et al. [28]	6	NS		2	4			
Zhou Y et al. [34]	8	3–5 (*n*=7) 10 (*n*=1)	1	1	4	2		
Total	100	3–15	25 (25%)	15 (15%)	29 (29%)	18 (18%)	4 (4%)	9 (9%)

Only articles detailing follow-up CT imaging are included in this table. The fzindings correspond to readily available reported findings within the relevant publications

*CT* computed tomography, *NS* not stated

### Imaging findings and demographics

Given the small number of cases and the heterogeneous nature of case reporting, we could not to determine whether differences in imaging presentations varied significantly with patient age group, gender or presenting symptoms.

Given the lack of available RT-PCR testing kits in many countries, there has been interest in using CT to identify early pulmonary findings suggestive of COVID-19, particularly where children are asymptomatic but at risk of disease because of infected co-habitants. In a subset of 30 children across 10 publications, we extracted imaging findings from asymptomatic children (Table S7) [5, 15, 16, 18, 19, 21, 23–27, 30–32, 34]. Of these, 11/30 (36.7%) had normal CT findings, 14/30 (46.7%) reported the more typical pattern of ground-glass opacification and 4/30 (13.3%) described nonspecific, consolidative changes, or “patchy shadowing”.

Regarding differences with adults, Ma et al. [23] found that children in their cohort (compared to a published adult cohort of 1,099 cases [3]), were more likely to demonstrate abnormalities on chest CT (86% [43/50] vs. 76% [840/1,099]), although these were less likely to be bilateral (18% [9/50] vs. 46% [505/1,099]) and less likely to demonstrate interstitial abnormalities (6% [3/50] vs. 13% [143/1,099]). The presence of ground-glass opacification (58% [29/50] vs. 50% [550/1,099]) and local patchy “shadowing” (32% [16/50] vs. 37% [409/1,099]) was similar between children and adults.

### Patient outcomes

At time of publication, 296/431 (68.7%) children had been discharged from the hospital, 72/431 (16.7%) were in a stable condition in a hospital, 3/431 (0.7%) had been admitted to intensive care units. One child admitted to intensive care reportedly later died at 4 weeks post hospital admission; this was a 10-month-old with multiorgan failure, septic shock and intussusception [5]. The outcome of the remainder of cases was unclear from the published reports (60/431, 13.9%).

One study performed subgroup analysis to determine the relationship between patient outcomes and CT imaging results [23]. The authors reviewed a subset of 23/50 (46.0%) children in their cohort who had been discharged, whose symptoms had resolved and who had negative RT-PCR testing on two separate occasions. The majority of these children 17/23 (73.9%) still demonstrated lung abnormalities, and in 2 cases these had progressed. A Cox regression analysis did not find any statistically significant association between differences in imaging findings during treatment and likelihood of discharge. This was supported in part by findings by Xia et al. [28], who stated that CT findings appeared to lag behind resolution of clinical symptoms and two sets of negative nucleic acid testing.

## Discussion

Chest CT imaging findings in children with COVID-19 are frequently normal or mild, with lower lobes most commonly affected, demonstrating patchy ground-glass opacification or, less frequently, areas of consolidation. Imaging appearances at follow-up remain normal or improve in the majority of children.

These findings raise important clinical implications for paediatricians and radiologists. Given the mild and sometimes absent findings on chest radiography and CT, it is unlikely that imaging will provide an increased confidence in the diagnosis for COVID-19, nor can it provide reassurance for the absence of infection if imaging is normal. This finding is supported by a recent study of 24 asymptomatic carriers of COVID-19, of whom 6 were children (5–15 years old) and all had normal chest CT findings [19]. Similar results have also been supported by work in adults [40, 41]. Whilst it is well known that chest radiography can underplay the chest CT findings, the identification of mild to moderately severe imaging appearances in the majority of children, with little subsequent change in management, is unlikely to justify the CT imaging.

Conversely, even when CT findings are present, we have found that these can be heterogeneous and nonspecific, and in the adult literature it has been reported that differentiation between COVID-19 and other viral infections based on imaging findings is difficult [42]. Research on this topic in paediatric radiology is currently unavailable, although Qiu et al. [30] compared clinical findings between children with COVID-19, H1N1 influenza and severe acute respiratory syndrome (SARS) and found that children with COVID-19 had a much milder course of illness than with SARS, but were more likely to develop pneumonia than those with H1N1 influenza. Future studies that focus on attempting to differentiate imaging findings among these viral illnesses in children should be mindful to account for co-existing viral infections given that one publication in this study demonstrated COVID-19 in a child with co-infection of RSV [22].

In recent publications, it has been shown that some paediatric patients might be “super spreaders” (i.e. high viral shedding) [43] and more likely to demonstrate early symptoms. Whether they also display earlier CT changes, which could be used to screen for individuals prior to available RT-PCR testing, remains unknown. Therefore, given the lower severity of disease and additional radiation burden, CT imaging should not be routinely conducted for diagnosis, but rather reserved for those with severe or deteriorating symptoms, or in the search for an alternative diagnosis to aid management.

The fact that imaging appearances frequently resolve, improve or remain normal at follow-up imaging is reassuring because it suggests that long-term pulmonary damage is unlikely, although at present there is insufficient evidence to confirm this. It is also important to bear in mind that the persistence of pulmonary findings does not necessarily imply ongoing infection, given that one study found persisting CT findings in 17/23 (73.9%) children who had been treated, with resolution of their symptoms and two negative RT-PCR tests. Therefore, follow-up CT imaging would also be better guided by clinical symptoms rather than being performed as a matter of routine. These recommendations have recently been incorporated into newly published guidelines for imaging in children with COVID-19 by the Radiological Society of North America (RSNA) [44], which encourages a pragmatic approach based on patient symptom severity using chest radiography for initial workup and only for follow-up in moderate to severe cases, reserving an initial chest CT only where a clinical change in management is anticipated and for the post-recovery stages. Useful advice regarding general staff protection and departmental organisation for imaging of COVID-19 cases has also been recently provided by the European Societies of Radiology (ESR) and Thoracic Imaging (ESTI) [45].

It is worth noting that although our inclusion criteria were not set to review infected pregnant women, there was one case in this review of an infected newborn, diagnosed at 36 h of age from a COVID-19-positive mother [27]. The authors proposed the possibility for vertical transmission as a route of infection; however, several subsequent articles reviewing outcomes of infected pregnant women have now suggested this to be unlikely [39, 46–48]. A recently published rapid review of coronavirus in pregnancy [49] found that of the 32 infected women identified in the literature, there was 1 stillbirth (34 weeks of gestation) and 15/32 (47%) pre-term deliveries. In the 15/32 (47%) neonates who were tested for COVID-19, all were negative. The latest guidance from the Royal College of Obstetricians and Gynaecologists [50] has therefore recommended against routine separation of affected mothers and their babies and has not found any evidence to suggest intrauterine fetal infection or teratogenic effects from the novel coronavirus. Clinicians should thus remain alert to alternative, more common diagnoses in newborns presenting with respiratory symptoms (e.g., respiratory distress syndrome, aspiration, pneumonia from alternative organisms), even if the mother is COVID-19 positive [48].

Although we performed a comprehensive systematic review, there are still several gaps in our radiologic knowledge regarding COVID-19 in children. In this article we included manuscripts relating to radiographic or CT appearances of lung pathologies in children, although information on other modalities is lacking. There is sparse literature on the use of portable point-of-care ultrasound (POCUS) for adult COVID-19 patients in Italy [51] and in children [52, 53]. In adults, POCUS has been reportedly used to triage more severe cases for urgent management by helping to identify areas of ground-glass opacification (so-called B lines) as well as areas of necrotic lung — a marker of the more advanced stages of infection [54]. Nevertheless, given that severe disease in children is less likely, the extent to which POCUS might be helpful is questionable, although it has been recommended as one of several potential options for lung assessment by a Chinese expert consensus review for neonatal management in COVID-19 [55]. Two adult publications have reported the use of [F-18]2-fluoro-2-deoxyglucose (FDG) positron emission tomography (PET)/CT in COVID-19 [56, 57], and suggested that it could help monitor disease progression and treatment outcomes, particularly by detecting residual activity in mediastinal lymph nodes. This modality has not been reported in infected children, and adult studies have only included a small number of cases (4 patients or fewer). The added value is thus still undetermined [58] and should not be first studied in children, particularly given the increased radiation burden.

Our review also did not find many articles reporting imaging findings in immunocompromised children, so it is undetermined whether these features might differ from those without health conditions. It has been well documented that more severe infections are found in immunocompromised children with other strains of coronavirus [59] and that these infections can spread to other parts of the body, such as the brain, causing encephalitis [60]. Whether this also occurs with COVID-19 remains to be seen. It is interesting that the only child death reported in this review was also suffering from an intussusception. Whether this was triggered by the underlying viral infection is unclear, but it is worth noting that gastrointestinal complaints can be the first and more prominent symptom of COVID-19 in some people and has been reported in an adult series [61].

There are several limitations to this work. Given the widespread public health interest, several manuscripts on the topic of COVID-19 are emerging each week, many bypassing usual peer review processes. It is likely that some information might be missing but later included, or in certain cases articles retracted (as for two articles during our screening process). Nevertheless, where possible, we have tried to mitigate this by conducting our literature review thrice in order to provide the most up-to-date information from reliable sources. Whilst not all imaging findings in all cases were reported in the studies, we described all available findings to give a general overview of the imaging pathology. Future works on the study of COVID-19 imaging findings could be improved by the use of standardised detailed descriptors for imaging findings (i.e. stating both the pattern and localisation of findings), in line with RSNA guidance [44], with clearly stated indications for imaging where possible.

Second, because of the origin of the virus in China, some articles have been published in a language other than English, or in English by non-native speakers. This might have hampered our understanding and interpretation of the data, although we used online translation services where required. Whilst other systematic reviews on the topic of COVID-19 have excluded articles not written in English, we thought it was important to review as many foreign-language articles where possible to increase our collective knowledge base, particularly given the few reported paediatric cases.

Finally, the majority of articles have included children from China, in particular Wuhan city. It is unclear whether some of these paediatric cohorts overlap, although we did identify three papers where there was clear similarity in many of the patients described, and we avoided repetition of findings in summary results. It is also important to highlight that differences in indications for CT imaging in children might also exist (which could explain why Ma et al. [23] found slightly more abnormalities on CT in children than adults), but unfortunately these indications were not made clear in the publications.

## Conclusion

Chest imaging findings in children with COVID-19 are frequently normal or mild, with unilateral changes that include patchy ground-glass opacification, commonly affecting the lower lobes. Imaging appearances at follow-up frequently remain normal or improve in the majority of children. Chest CT imaging adds little to the further management of the child and should be reserved for severe cases or for identifying alternative diagnoses. Further areas of research should include information on imaging and clinical characteristics in immunocompromised children with COVID-19, and information on long-term follow-up, particularly in the more severely affected children. We should therefore be prudent with the usage of CT, particularly at a time where resources are stretched, and only use it in the more vulnerable populations.

## Electronic supplementary material

ESM 1 (DOCX 47.3 kb)
