# Supplementary material for: Coronavirus disease 2019 (COVID-19) in children: a systematic review of imaging findings
Source: Pediatr Radiol. 2020 Jun 18;50(9):1217–30. doi: 10.1007/s00247-020-04726-w (PMC7300372; doi:10.1007/s00247-020-04726-w)
Supplement: Supplementary file 1 — (DOCX 47.3 kb) [file 247_2020_4726_MOESM1_ESM.docx]

**Electronic Supplementary Material**

**Table S1** Medline search strategy, 2015–2020 = 97 hits

| **MEDLINE** | **Search terms** |
| --- | --- |
| #1 | "COVID-19" [all fields] OR "2019-nCoV" [all fields] OR "2019nCoV" [all fields] OR "coronavirus" [all fields] |
| #2 | "Pediatrics" [MeSH] OR "Child" [MeSH] OR "Adolescent" [MeSH] OR child* OR adolescen* OR teen* OR youth [tiab] OR young [tiab] OR pediatric* [tiab] OR paediatric* [tiab] |
| #3 | "Radiology" [MeSH terms] OR "radiology" [all fields] OR "radiography" [MeSH terms] OR "radiography" [all fields] OR "imaging" [all fields] OR “CT” [all fields] OR “CT” [MeSH terms] OR “MRI” [all fields] OR “MRI” [MeSH terms] OR “Ultrasound” [all fields] OR “Ultrasound” [MeSH terms] OR “Ultrason*” [all fields] OR “Ultrason*” [MeSH terms] |
| #4 | # 1 AND #2 AND #3 |

*MeSH* medical subject headings

**Table S2** Embase search strategy, 2015–2020

| **#** | **Searches** | **Results** |
| --- | --- | --- |
| 1 | (coronavirus or covid-19 or nCoV).af | 4929 |
| 2 | exp pediatrics/ or child/ or adolescent/ or (child* or adolescen* or teen* or youth or young or pediatric* or paediatric*).af | 902463 |
| 3 | (radiology/ or imaging or ultrason* or sono* or CT or radiograph* or magnetic resonance).af | 2807057 |
| 4 | 1 and 2 and 3 | 60 |

**Table S3** Cochrane Library search strategy = 0 hits

|  | **Search terms** |
| --- | --- |
| #1 | Coronavirus or COVID-19 or nCoV or 2019nCoV |
| #2 | child* or adolescen* or teen* or youth or young or pediatric* or paediatric*:ti,ab,kw (Word variations have been searched) |
| #3 | radiology or imaging or radiograph* or ultraso* or magnetic res* or CT:ti,ab,kw (Word variations have been searched) |
| #4 | # 1 AND #2 AND #3 |

**Table S4** Study details and patient characteristics from studies of patients with COVID-19

| **Author [reference]** | **City, country** | **Article language** | **Sample size^a^** | | **Study design** | **Time frame (DD/MM/YY)** | **Gender** | | **Mean age (range)** | **Presenting symptoms** | |
| --- | --- | --- | --- | --- | --- | --- | --- | --- | --- | --- | --- |
|  |  |  | **Adult** | **Child** |  |  | **M** | **F** |  | **None** | **Symptoms^b^** |
| Cai J et al. [14] | Shanghai, China | English | 0 | 10 | RCS | 19/01/20 – 03/02/20 | 4 | 6 | 6.2 y  (3 m – 10.9 y) | - | 8 (80%) fever  6 (60%) cough  4 (40%) sore throat  3 (30%) stuffy nose  2 (20%) rhinorrhoea |
| Chan JF et al. [15] | Shenzhen, China | English | 5 | 1 | RCS | 29/12/19 – 04/01/20 | 1 | 0 | 10 y | 1 (100%) | - |
| Chen C et al. [16] | Shenzhen, China | English | 0 | 31 | PCS | 16/01/20 – 19/02/20 | 13 | 18 | 7.3 y  (1.5–17 y) | 12 (38.7%) | 14 (45.2%) fever  13 (41.9%) cough |
| Cui Y et al. [17] | Guizhou, China | English | 0 | 1 | CR | 28/01/20 | 0 | 1 | 55 d | - | 1 (100%) dry cough, rhinorrhoea |
| Feng K et al. [18] | Shenzhen, China | Chinese | 0 | 15 | RCS | 16/01/20 – 06/02/20 | 5 | 10 | 7 y  (4–14 y) | 10 (66.7%) | 5 (33.3%) fever |
| Hu Z et al. [19] | Nanjing, China | English | 18 | 6 | RCS | 28/01/20 – 09/02/20 | 3 | 3 | 9.7 y  (5–15 y) | 6 (100%) (1 10-y female patient developed fever later) | - |
| Ji LN et al. [20] | Beijing, China | English | 0 | 2 | RCS | 25/01/20 – 03/02/20 | 2 | 0 | 12 y  (9–15 y) | - | 1 (50%) fever  1 (50%) diarrhoea |
| Li W et al. [21] | Zhuhai, China | English | 0 | 5 | RCS | 28/01/20 – 08/02/20 | 4 | 1 | 3.4 y  (10 m – 6 y) | 4 (80%) | 1 (20%) cough + fever |
| Liu H et al. [22] | Shanghai, China | English | 55 | 4 | RCS | 27/01/20 – 14/02/20 | 2 | 2 | 3.8 y  (2 m – 9 y) | - | 3 (75%) fever  3 (75%) cough  1 (25%) fatigue |
| Liu M et al. [31] | Chongqing, China | English | 0 | 5 | RCS | NS | 4 | 1 | 6 y  (7 m – 13 y) | 3 (60%) | 2 (40%) fever  2 (40%) cough |
| Lu X et al. [5] | Wuhan, China | English | 0 | 171 | RCS | 28/01/20 – 26/02/20 | 104 | 67 | Median 6.7 y  (1 d – 15 y) | 27 (15.8%) | 33 (19.3%) URTI  111 (64.9%) Pneumonia |
| *Sun D et al.^c^* [12]  *(Subgroup population of Lu X et al)* | *Wuhan,*  *China* | *English* | *0* | *8* | *RCS* | *24/01/20 – 24/02/20* | *6* | *2* | *6.8y*  *(2m – 15y)* | *-* | *8 (100%) polypnea*  *6 (75%) fever*  *6 (75%) cough*  *4 (50%) nausea*  *3 (38%) diarrhoea* |
| *Ma YL et al.^c^* [13]  *(Subgroup population of Lu X et al)* | *Wuhan, China* | *Chinese* | *0* | *115* | *RCS* | *NS* | *73* | *42* | *51 d – 15 y*  *Mean/median age not provided* | *61 (53%)* | *29 (25.2%) fever*  *47 (40.9%) URTI*  *5 (4.3%) other — including 3 with gastrointestinal sympotms, 2 with chest pain* |
| Ma H et al. [23] | Wuhan,  China | English | 0 | 50 | RCS | 21/01/20 – 14/02/20 | 28 | 22 | 2.5 y  (0.9–7.0 y) | 6 (12%) | 32 (64%) fever  22 (44%) cough  6 (16%) rhinorrhoea  3 (6%) diarrhoea  2 (4%) abdominal pain  2 (4%) myalgia  1 (2%) sore throat  1 (2%) intussusception |
| Pan X et al. [24] | Guangzhou,  China | English | 2 | 1 | RCS | 27/01/20 – 29/01/20 | 1 | 0 | 3 y | 1 (100%) | - |
| Park JY et al. [25] | Seoul,  S. Korea | English | 0 | 1 | CR | 18/02/20 | 0 | 1 | 10 y | 1 (100%) initially (developed fever 13 days after COVID-19 exposure) | - |
| Qiu H et al. [30] | Ningbo, China | English | 0 | 36 | RCS | 17/01/20 – 01/03/20 | 23 | 13 | 8.3 y  (1–16 y) | 10 (28%) | 17 (19%) cough  13 (36%) fever  3 (8%) headache  2 (6%) sore throat  2 (6%) vomiting or diarrhoea  1 (3%) dyspnoea |
| Rahimzadeh G et al. [29] | Iran (multiple cities) | English | 0 | 9 (only 3 were PCR+ and included in this review) | RCS | NS | 3 | 0 | 4.3 y  (3–5 y) | - | 3 (100%) fever  3 (100%) cough  3 (100%) tachypnoea |
| Shen Q et al. [32] | Hunan,  China | English | 0 | 9 | RCS | 08/01/2020 – 19/02/2020 | 3 | 6 | 8 y  (1–12 y) | 2 (22.2%) | 3 (33.3%) fever  2 (22.2%) diarrhoea  1 (11.1%) cough  1 (11.1%) sore throat |
| Tang A et al. [26] | Shenzhen,  China | English | 0 | 26 | RCS | 16/01/20 – 08/02/20 | 9 | 17 | 6.9 y  (1–13) | 9 (34.6%) | 11 (42.3%) fever  11 (42.3%) cough  2 (7.7%) rhinorrhoea  2 (7.7%) diarrhoea  2 (7.7%) vomiting |
| Wang S et al. [27] | Wuhan, China | English | 0 | 1 | CR | 01/02/20 | 1 | 0 | 36 hours (term pregnancy) | 1 (100%) (mother infected during pregnancy) | - |
| Xia W et al. [28] | Wuhan,  China | English | 0 | 20 | RCS | 23/01/20 – 08/02/20 | 13 | 7 | Median 2y1m  (1 d – 14y7m) | - | 12/20 (60%) fever  13/20 (60%) cough  3 (15%) diarrhoea  3 (15%) rhinorrhoea |
| Zheng F et al. [33] | Hubei province (3 centres), China | English | 0 | 25 | RCS | 01/02/2020 – 10/02/2020 | 14 | 11 | Median 3 y  (3 m – 14 y) | - | 13 (52%) fever  11 (44%) cough  3 (12%) diarrhoea  2 (8%) nasal congestion  2 (8%) dyspnoea  2 (8%) vomiting |
| Zhou Y et al. [34] | Shenzhen, China | Chinese | 0 | 9 | RCS | 20/01/2020 – 10/02/2020 | 4 | 5 | Median 1 y  (0–3 y) | 5 (55.6%) | 4 (44.4%) fever  2 (22.2%) cough  1 (11.1%) rhinorrhoea |

*CR* case report, *CS* case series, *CT* computed tomography, *CXR* chest radiography, *d* days, *DD/MM/YY* day/month/year, *F* female, *m* months, *M* male, *NR* not reported, *NS* not stated, *PCS* prospective case series, *RCS* retrospective case series, *URTI* upper respiratory tract infection, *y* years

^a^ Sample size includes numbers of adults and children included in each study cohort; however gender/age/clinical details only pertain to the paediatric cases

^b^ Symptoms are non-mutually exclusive. Only laboratory test confirmed COVID-19 (using reverse transcriptase polymerase chain reaction) reported

^c^ High likelihood of overlapping data between studies. Information listed here for reference only, not included in later summative analysis

**Table S5** Imaging characteristics of children with COVID-19

| **Author**  **[reference]** | **Sample size (children)** | **Imaging modality** | **Initial imaging timing^a^** | **Initial imaging findings^b^** | **Follow-up imaging date** | **Follow-up imaging findings^b^** | **Patient outcome**  **(and co-morbidities, if present)** |
| --- | --- | --- | --- | --- | --- | --- | --- |
| Cai J et al. [14] | 10 | CXR | Admission | 6 (60%) normal  4 (40%) unilateral patchy infiltrates  (1 retrocardiac opacity, left lung; 3 right lung opacification) | None | None | All discharged within same month without any complications |
| Chan JF et al. [15] | 1 | Chest CT | NS | CT showed GGO (location and laterality not mentioned) | None | None | Stable condition, still inpatient at time of report (9 days post admission) |
| Chen C et al. [16] | 31 | Chest CT | Admission | 20 (64.5%) normal  8 (25.8%) unilateral pneumonia  3 (9.7%) bilateral pneumonia  Example imaging in 4 cases all demonstrate patchy peripheral GGO in middle or lower lobes. | NS | - 19 (61.3%) remained normal  - 8 (25.8%) unilateral pneumonia  (1 previously normal,  7 unchanged unilateral pneumonia)  - 1 (3.2%) bilateral pneumonia (previously unilateral)  - 3 (9.7%) no follow-up CT mentioned | 23 (74.2%) discharged at time of report  8 (25.8%) stable condition, remain inpatients |
| Cui Y et al. [17] | 1 | Chest CT | Admission (Day 6 post symptoms) | Mild perihilar GGO in RUL and RLL | Chest CT Day 4 and Day 11 post admission (Days 9 and 16 post symptoms) | - CT Day 4: progression of perihilar, RUL, RLL GGO  - CT day 11:  Resolution of GGO. RUL linear atelectasis only | Asymptomatic and still in isolation at an external hospital on Day 25 post admission at time of report |
| Feng K et al. [18] | 15 | Chest CT | Admission | 6 (40%) normal  9 (60) inflammatory infiltrations – patchy nodular, GGO, visible halo sign (4 in single lobar segment; 4 in at least two lobar segments; 1 in more than two lobar segments) | 3–5 days post admission | - 2 (13.3%) resolution of previous GGO  - 7 (46.7%) stable GGO  - 3 (20%) remain normal  - 3 (20%) new GGO from normal CT before | 5 (33.3%) cases discharged after average stay of 12 days (7–17 days)  10 (66.7%) stable inpatients at time of report |
| Hu Z et al. [19] | 6 | Chest CT | Admission | - 4 (66.7%) normal  - 1 (16.7%) right basal subpleural ground-glass opacification  - 1 (16.7%) ground-glass opacification/patchy shadowing | 13 days post admission | Case of right basal opacification resolved  Other cases not stated | 3 (50%) discharged (after 5–7 days of admission)  3 (50%) stable inpatients at time of report (admission time 11–21 days) |
| Ji LN et al. [20] | 2 | Chest CT | NS | 2 (100%) normal | None | None | Symptoms resolved after 2 days of treatment for both cases |
| Li W et al. [21] | 5 | Chest CT | 4 days post admission  (range 2–9 days) | - 2 (40%) normal  - 3 (60%) patchy GGO  (2 LLL, 1 RUL) | 5–7 days post first CT | 3 (60%) had follow-up imaging, all normal | 3 (60%) discharged home (12–14 days after admission)  2 (40%) remained in hospital at 13 and 24 days post admission at time of publication |
| Liu H et al. [22] | 4 | Chest CT | NS, presumed on admission | - 1 (25%) normal  - 1 (25%) single area of consolidative change, RLL  - 1 (25%) single area of GGO, RML  - 1 (25%) multifocal consolidation + pleural effusion | 7 days post admission | 1 (25%) Remained normal  2 (50%) Improving appearances of consolidative and GGO change  Child with multifocal involvement not re-imaged | No information regarding discharge or length of stay for paediatric patients  Co-morbidities: 1 child with multifocal consolidation and effusion was infected with both COVID-19 and RSV |
| Liu M et al. [31] | 5 | Chest CT | Admission | - 1 (20%) normal  - 2 (40%) unilateral GGO  - 1 (204%) unilateral GGO and consolidation  - 1 (20%) bilateral GGO | 4–14 days post admission | 1 (20%) remained normal  4 (80%) had complete resolution | No information regarding discharge or length of hospital stay; however, all children demonstrated normal CT findings at 4–14 days post diagnosis |
| Lu X et al. [5] | 171 | Chest CT | NS | - 60 (35%) none  - 56 (32.7%) ground-glass opacification  - 32 (18.7%) local patchy shadowing  - 21 (12.3%) bilateral patchy shadowing  - 2 (1.2%) interstitial abnormalities | NS | NS | 1 death (10-month-old, multiorgan failure, intussusception) died 4 weeks after admission  3 were admitted to ITU and had co-morbidities (1 leukaemia, 1 hydronephrosis, 1 intussusception)  149 (87.1%) discharged at time of publication |
| *Sun D et al.*^c^ [12]  *(Subgroup population of Lu X et al.)* | *8* | *CXR*  *Chest CT* | *NS, presumed on admission*  *CXR and CT findings described together* | *Location of findings:*  *- 2 (33.3%) unilateral pneumonia*  *- 6 (75%) bilateral pneumonia*  *Imaging description:*  *- 1 (12.5%) ‘white lung’(figure demonstrates bibasal opacification/consolidation)*  *- 1 (12.5%) multiple patchy GGO + pleural effusion*  *- 6 (75%) multiple, patchy GGO/shadows* | *Unclear* | *-* | *5 (62.5%) Discharged*  *3 (60%) Remain on intensive care unit at time of report*  *Co-morbidities:*  *1 (12.5%) Leukaemic patient (remained on intensive care unit at this report)*  *Complications during admission:*  *- 1 (12.5%) developed intussusception, toxic encephalopathy, septic shock and multi-organ dysfunction*  *- 1 (12.5%) septic shock, hydronephrosis, cardiac insufficiency*  *- 1 (12.5%) hypoglobulinaemia*  *- 1 (12.5%) gastroenteritis* |
| *Ma YL et al.*^c^  [13]  *(Subgroup population of Lu X et al.)* | *115* | *Chest CT* | *NS, presumed on admission* | *- 27 (23.5%) normal*  *- 49 (42.6%) GGO and consolidation*  *- 39 (33.9%) “enhanced lung texture” reported (interpreted as infiltrates)*  *- 2 (1.7%) “white lung”*  *- 2 (1.7%) pleural effusions* | *-* | *-* | *Outcome data not included*  Co-morbidities: *3 critically ill*  -13-month-old male with hydronephrosis, 8-year-old male with acute lymphoblastic leukemia, *11-month-old male with intussusception* |
| Ma H et al. [23] | 50 | Chest CT | NS, presumed on admission | - 29 (67%) ground-glass opacities  - 16 (37%) localized patchy shadowing  - 9 (21%) bilateral patchy shadows  - 3 (7%) interstitial lesions  - 1 (2%) pleural effusion  Location  - 28 (65%) lower lobe lesions  - 9 (18%) middle lobe  - 22 (44%) upper lobe | Repeat CT in 29 (58%) patients, time unspecified | 2/29 (7%) resolution  17/29 (58%) partially resolved  2/29 (7%) no change  8/29 (28%) worsening changes | 23 (46%) discharged at time of report |
| Pan X et al. [24] | 1 | Chest CT | 1 day post admission | Normal | NS | NS | NS |
| Park JY et al. [25] | 1 | CXR  Chest CT | Admission CXR and CT | CXR: Normal  CT: Patchy, nodular consolidations with peripheral GGO in subpleural areas of RLL | CXR 3 days later | Normal | Transferred for isolation at an external hospital after 15 days post admission, at time of report |
| Qiu H et al. [30] | 36 | Chest CT | NS | 17 (47%) Normal  19 (53%) GGO | None | None | No cormorbidities stated  Average duration of hospital stay was 14 days (10–20 days, range). All patients recovered, no deaths |
| Rahimzadeh G et al. [29] | 3 RT-PCR positive cases | CXR  Chest CT | CXR at admission  CT not specified, presumed upon admission also | CXR  - 2 (66.7%) ‘airspace shadowing’ (location not specified)  - 1 (33.3%) no findings recorded, uncertain if this was performed  CT  - 2 (66.7%) patchy consolidation with halo sign, and GGO in both lungs  - 1 (33.3%) normal | None | None | No comorbidities, none required intensive care  - 1 (33.3%) discharged at 3 days  - 2 (66.7%) had improvement in general condition (length of hospital stay unknown) |
| Shen Q et al. [32] | 9 | CXR  Chest CT (interpreted together) | NS | - 7 (77.8%) normal  - 2 (22.2%) unilateral GGO | None | None | None required intensive care admission nor ventilation  - 3 (33.3%) remained in hospital at time of publication  - 6 (66.7%) discharged (length of stay 11–22 days) |
| Tang A et al. [26] | 26 | CXR  Chest CT | NS | - 11 (42%) lateral pulmonary infiltrates  - 7 (27%) bilateral pulmonary infiltrates | NS | NS | - 17 (65%) discharged at 13.6 days (+/- 1.03 days)  - 9 (35%) remained in hospital at time of report |
| Wang S et al. [27] | 1 | CXR  Chest CT | CXR at Day 2 post admission  CT at Day 4 post admission | Initial CXR showed thickened lung texture  Initial CT showed high-density nodular shadow at posterior segment of LUL | Two further CT chest at Days 10 and 15 | Second CT on Day 10 showed LUL and LLL scattered GGO  Third CT on Day 15 showed patchy shadow in LUL, improved from second CT | Discharged home after 17 days of admission |
| Xia W et al. [28] | 20 | Chest CT | NS, presumed on admission | - 4 (20%) normal  - 6 (30%) unilateral pulmonary lesion  - 10 (50%) bilateral lung lesions  Of these,  - 16 (80%) subpleural ground-glass opacities  - 10 (50%) central consolidation with surrounding ground-glass halo  - 12 (60%) ground-glass opacities  - 4 (20%) fine mesh shadow  3 (15%) micronodules | Second CT in 6 (30%) patients, timing not stated | 2 (10%) compete resolution  3 (15%) consolidations partially resolved with residual ground-glass opacities  3 (15%) residual fibrous band | 18 (90%) discharged with mean length of stay of 12.9 days (8–20 days)  2 (10%) asymptomatic neonates still under observation with negative CT findings at time of report |
| Zheng F et al. [33] | 25 | Chest CT (in 24/25, 96% patients) | NS, presumed on admission | - 8 (33%) normal  - 5 (21%) unilateral findings  - 11 (46%) bilateral findings  - Number of cases with different pattern of abnormalities not stated, although typical findings of bilateral patchy shadows or consolidations were mentioned | - | - | Co-morbidities: 2 (8%) congenital cardiac disease (not specified); both required mechanical ventilation during hospital stay  At time of reporting, 1 (4%) patient had been discharged and the remainder (24/25, 96%) were improving in symptoms |
| Zhou Y et al. [34] | 9 | Chest CT | CT within 3 days of admission | Pattern  - 1 (11.1%) normal (incidental bullae noted in LLL)  - 8 (88.9%) inflammatory changes (6 GGO with consolidation; 1 consolidation only; 1 GGO only)  - 1 (11.1%) pleural effusion  - 3 (33.3%) halo sign  Distribution  - 4 (50%) bilateral  - 4 (50%) unilateral  Location  - 6 (75%) upper lobe  - 6 (75%) lower lobe  - 5 (62.5%) middle lobe | Second CT in 7/9 (77.8%) patients, after 3–5 days from previous imaging  Second CT in 1/9 (11.1%) children 10 days later | 1 (12.5%) remained normal  1 (12.5%) complete resolution  4 (50%) improvement  2 (25%) stable appearances of prior findings | At time of report, all children remained as inpatients, none were admitted to the intensive care unit  Co-morbidities not reported |

*ARDS* acute respiratory distress syndrome, *CXR* chest radiography, *GGO* ground-glass opacification, *ITU* intensive therapy unit, *LUL* left upper lobe, *LLL* left lower lobe, *NC* non-contrast, *RUL* right upper lobe, *RML* right middle lobe, *RLL* right lower lobe, *RSV* respiratory syncytial virus, *RT-PCR* reverse transcriptase polymerase chain reaction, *US* ultrasound

^a^ Initial timing of imaging might be days since onset of symptoms or days since admission to hospital

^b^ Imaging patterns described are as written in the publications

^c^ High likelihood of overlapping data between studies. Information listed here for reference only, not included in later summative analysis.

**Table S6** CT imaging protocols/parameters used in children in this systematic review

| **Author [reference]^a^** | **Radiologist**  **co-author** | **CT vendor** | **CT model** | **Intravenous contrast^b^** | **Parameters^c^** |
| --- | --- | --- | --- | --- | --- |
| Chan JF et al. [15] | N | NS | NS | N | NS |
| Chen C et al. [16] | N | NS | NS | N | NS |
| Cui Y et al. [17] | N | NS | NS | N | 80 kV, automatic tube current (23–26 mAs) |
| Feng K et al. [18] | Y | Toshiba | TSX-101A | N | 64-slice MDCT. 136 kV, automatic tube current (unspecified)  Matrix 612x612, FOV 320x320 mm  Slice thickness 6 mm. Slice interval 1 mm. Pitch 0.8  Reconstruction performed with lung window algorithm |
| Hu Z et al. [19] | Y | NS | NS | N | NS |
| Ji LN et al. [20] | N | NS | NS | N | NS |
| Li W et al. [21] | Y | NS | NS | N | NS |
| Liu H et al. [22] | Y | GE | Optima 660 | N | 64-slice MDCT. 100 kV, automatic tube current (30–100 mA)  Slice thickness 3–5 mm. Slice interval 1 mm. Rotation speed 0.6 s. Pitch 0.969:1  Reconstruction of images at 0.625 to 1-mm thickness with lung window algorithm |
| Liu M et al. [31] | Y | NS | NS | N | NS |
| Lu X et al. [5] | U | NS | NS | NS | NS |
| Ma H et al. [23] | Y | Siemens  GE | SOMATOM Definition AS128  Optima 660 | N | Slice thickness 0.625 mm |
| Pan X et al. [24] | Y | NS | NS | NS | NS |
| Park JY et al. [25] | Y | NS | NS | Y | NS |
| Qiu H et al. [30] | N | NS | NS | N | NS |
| Rahimzadeh G et al. [29] | N | NS | NS | N | NS |
| Shen Q et al. [32] | N | NS | NS | NS | NS |
| Tang A et al. [26] | N | NS | NS | NS | NS |
| Wang S et al. [27] | N | NS | NS | N | NS |
| Xia W et al. [28] | Y | Siemens | SOMATOM Definition AS128 | N | 120 kV, automatic tube current (100 to 150 mA)  Collimation 0.6 mm. Pitch 1:1  CT images were reconstructed with 1.25‐mm collimation with lung window algorithms |
| Zheng F et al. [33] | N | NS | NS | NS | NS |
| Zhou Y et al. [34] | Y | Toshiba | Toshiba TSX-101A | N | 80–120 kV, automatic tube current  FOV 200x200 mm  Collimation 0.8 mm with lung window algorithms |

*FOV* field of view, *MDCT* multidetector computed tomography, *N* no, *NS* not stated, *U* uncertain, *Y* yes

^a^ Articles describing or using only chest radiography have been excluded from this table

^b^ Where the usage of intravenous contrast agent for CT imaging is not described, this was inferred from imaging included as figures from the manuscript

^c^ Given wide differences in availability of reported CT imaging parameters, the full descriptions as stated in the article are listed here

**Table S7** CT imaging characteristics of asymptomatic children with COVID-19

| **Author**  **[reference]** | **Article language** | **Sample size^a^** | | **Asymptomatic**  **child(ren)** | **Imaging findings in asymptomatic child(ren)^b^** |
| --- | --- | --- | --- | --- | --- |
|  |  | **Adult** | **Child** |  |  |
| Chen C et al. [16] | English | 0 | 31 | 12 | Not possible to extract |
| Chan JF et al. [15] | English | 5 | 1 | 1 | 1 GGO (location and laterality not mentioned) |
| Feng K et al. [18] | Chinese | 0 | 15 | 10 | Not possible to extract |
| Hu Z et al. [19] | English | 18 | 6 | 6 | 4 normal  1 right basal subpleural GGO  1 GGO/patchy shadowing |
| Li W et al. [21] | English | 0 | 5 | 4 | 2 normal  2 patchy GGO (1 LLL, 1 RUL) |
| Liu M et al. [31] | English | 0 | 5 | 3 | 1 normal  1 unilateral GGO  1 bilateral GGO |
| Lu X et al. [5] | English | 0 | 171 | 27 | Not possible to extract |
| Ma H et al. [23] | English | 0 | 50 | 6 | 2 normal  2 GGO  2 localized patchy shadowing |
| Pan X et al. [24] | English | 2 | 1 | 1 | 1 normal |
| Park JY et al. [25] | English | 0 | 1 | 1 | 1 patchy, nodular consolidation with peripheral GGO in subpleural areas of RLL |
| Qiu H et al. [30] | English | 0 | 36 | 10 | Not possible to extract |
| Shen Q et al. [32] | English | 0 | 9 | 2 | 2 normal |
| Tang A et al. [26] | English | 0 | 26 | 9 | Not possible to extract |
| Wang S et al. [27] | English | 0 | 1 | 1 | 1 high-density nodular shadow at posterior segment of LUL |
| Zhou Y et al. [34] | Chinese | 0 | 9 | 5 | 1 normal  1 bilateral GGO with halo sign  1 unilateral GGO with consolidation and effusion  1 unilateral GGO with consolidation  1 unilateral GGO only |

*CT* computed tomography, *GGO* ground-glass opacification, *LUL* left upper lobe, *LLL* left lower lobe, *RUL* right upper lobe

^a^ Sample size includes numbers of adults and children included in each study cohort; however gender/age/clinical details only pertain to the paediatric cases

^b^ Symptoms are non-mutually exclusive. Only reverse transcriptase polymerase chain reaction (RT-PCR) positive patients reported
